# S07-3: Exploring association between characteristics of green space and HEPA behaviour of teenagers in Slovenia

**DOI:** 10.1093/eurpub/ckae114.229

**Published:** 2024-09-26

**Authors:** Ina Šuklje Erjavec, Jana Kozamernik, Vita Žlender

**Affiliations:** Urban Planning Institute of the Republic of Slovenia, Ljubljana, Slovenia; Urban Planning Institute of the Republic of Slovenia, Ljubljana, Slovenia; Urban Planning Institute of the Republic of Slovenia, Ljubljana, Slovenia

## Abstract

**Purpose:**

Different characteristics of public green areas are important spatial conditions for sustainable development as well as for promotion and quality of everyday physical activity (PA). However, not all green places are suitable for all kinds of PA and different people have different needs, preferences and motivations that should be considered for suitable provision of the sustainable and inclusive environment for HEPA. One of the less investigated and thus understood population groups is teenagers. Their needs and motivations as well as behaviour patterns are often overlooked when researching and planning for quality of living environment although there is a growing concern about their physical inactivity and sedentary lifestyles.

**Methods:**

This study focuses on the role of physical environment in elevating PA. It questions how we can plan outdoor green spaces to create a safe and quality environment to sustain and encourage PA among teenagers. It aims to provide answers by (1) inspecting the needs, wishes and preferences of teenagers regarding PA and, (2) identifying facilitators and barriers to outdoor PA. A national-wide survey with 165 respondents and two workshops with more than 100 high school participants were used to collect data from teenagers in Slovenia.

**Results:**

Respondents’ main activities were walking, socializing outdoors, and hiking. However, 70% of them did not reach sufficient PA, mainly due to the lack of time and company for PA. Whilst a recreational paths network and a space to play ball were the main encouragements for outdoor PA, crowdedness, rain, bad smell and trash were among main barriers.

**Conclusions:**

Our results suggest that young people appreciate similar green space characteristics to other population groups as secure access and use, natural shade, closeness to home, and trees. But there are some specific aspects as playing ball settings, possibilities for socializing and the evening use, WI-FI provision and similar that should be taken into consideration. However, we can conclude that providing large, clean, and well-maintained semi-natural or natural-like green spaces close to people's homes may be an effective strategy to improve PA and youth’s health.

**Funding Source:**

Slovenian Research and Innovation agency (ARIS).

